# Accuracy of the Horizontal Calibrator in Correcting Leg Length and Restoring Femoral Offset in Total Hip Arthroplasty

**DOI:** 10.3389/fsurg.2022.845364

**Published:** 2022-03-03

**Authors:** Xing Chen, Shuxing Xing, Zhiyong Zhu, Huisheng Wang, Zhongshen Yu, Xizhuang Bai, Xi Li

**Affiliations:** ^1^Department of Orthopedic Surgery, Chengdu Fifth People's Hospital, The Fifth People's Hospital of Chengdu University of TCM, Chengdu, China; ^2^Department of Orthopedics and Sports Medicine and Joint Surgery, Liaoning Provincial People's Hospital, People's Hospital of China Medical University, Shenyang, China

**Keywords:** hip arthroplasty, leg length discrepancy, offset, intraoperative, calibrator

## Abstract

**Background:**

Limb length discrepancy (LLD) is one of the most common postoperative complications and can cause serious consequences. Poor recovery of femoral offset (OD) will result in weakness of the patient's external rotator muscles and affect the patient's postoperative function. The study is aimed to present a simple approach that compensates for the shortcomings of previous measuring devices and combines the advantages of different measuring devices to provide more accurate limb length and femoral offset restoration in total hip arthroplasty (THA).

**Methods:**

This study was a prospective controlled trial involving 89 patients with THA. Group I (*n* = 44) was used for intraoperative measurement of THA with our self-designed horizontal calibrator. Group II (*n* = 45) was measured by a traditional freehand technique. The main outcome indicators were measured on the Neusoft PACS, including LLD, femoral offset deviation, and operative time. IBM SPSS 23.0 was used for data analysis.

**Results:**

The independent sample *t-*test was performed for all the data. The operative time, preoperative radiographic LLD, and OD of Group I and Group II had no statistical significance. Postoperative LLD of Group I and Group II were 2.5 ± 2.1 mm (range −5.7 to 8.3 mm) and 6.2 ± 4.3 mm (range −18.0 to 15.2 mm), and the independent sample *t-*test data of both (*P* < 0.001; 95% CI = −5.1, −2.2) showed statistical significance. In Group I, there were 38 THAs with LLD <5 mm, accounting for 86% and there were 44 THAs with LLD <10 mm, accounting for 100%. In Group II, there were 20 THAs with LLD <5 mm, accounting for 44%. There were 36 THAs with LLD <10 mm, covering for 80%. There was no significant difference in postoperative femoral offset and OD.

**Conclusion:**

The horizontal calibrator can provide more accurate limb length and femoral offset recovery in THA. It is a simple surgical technique that does not add additionally surgical costs and does not significantly increase operative time, providing a new solution for surgeons to resolve postoperative LLD and restore femoral offset.

## Introduction

Total hip arthroplasty (THA) is the most successful and cost-effective orthopedic surgery for patients with end-stage hip arthritis that relieves pain, restores function and improves quality of life (1). Limb length discrepancy (LLD) is one of the most common causes of lawsuit after THA in the United States (2). LLD may lead to biomechanical changes in the hip joint, gait dysfunction, low back pain, sciatica, instability, and increased risk of dislocation (3). The incidence of LLD after primary THA has been reported to be 1–27%, with an average of 3–17 mm and a range of 3–70 mm (1, 4). The research results of Fujita et al. (5) showed that 7 mm might be a reasonable threshold to reduce residual discomfort.

Although preoperative and postoperative LLD can be reliably measured by clinical examination and radiographs, intraoperative assessment of LLD is difficult (4). Various measurement techniques have been used to evaluate limb length intraoperatively. The freehand technique is a widely used technique, but it reveals great interobserver and intraobserver variability (6). Preoperative templates are also widely used, and digital templates have emerged to make the operation more convenient and the results more accurate (7, 8). But studies have shown that in up to 60% of the cases, the preoperative template cannot accurately predict the correct size of the implant (9). Some studies have also reported the use of intraoperative radiography to assess limb length and offset, but the postural requirements are greatly high (10, 11). Intraoperative navigation may yield satisfying results, but its application is limited by the difficulty of finding anatomical navigation points in obese patients and the high price (6). Also some studies reported no difference in leg length balance between computer-assisted and conventional THAs (1). Most surgeons use a variety of devices to accurately measure the length of the neck and the angle of the osteotomy, as well as devices that are attached to the pelvis to determine changes in the length of the implant after it has been placed (12, 13). However, there are limitations in obese patients, and the complications of pelvic fixation have been reported (3, 14).

The mechanical relationship between the abductor tissue and the direction of the femur is known as femoral offset (OD). OD is the distance from the center of the femoral head to the shaft axis of the femoral component. Inadequate reduction of OD results in decreased abductor tension and subsequent instability, thereby affecting gait symmetry. In addition, inappropriate OD may increase the risk of instability due to bone impingement. Fackler and Pose found that femoral displacement was significantly reduced in patients with postoperative dislocation and concluded that lateral rather than distal femoral stalk displacement enhanced stability. Since OD recovery is not physically obvious to patients, it receives less attention intraoperatively than LLD, and therefore less intraoperatively verified (15, 16).

The method based on the change of the position of the reference point of the pelvis and femur is an effective way to minimize LLD and OD recovery. Although there are many devices designed according to this idea at the present such as: Double-Stitch Technique (17), L-shaped caliper (18), LOOD device (14), calipers dual pin retractor (19), they also have obvious disadvantages, such as susceptibility to changes in limb position, complex operation, poor measurement accuracy, inconsistent anchor points, muscle contracture, and soft tissue effects (1, 3, 4). This article proposes a simple method that compensates for the shortcomings of previous measuring devices (12, 13, 16, 17, 19–23) and combines the advantages of different measuring instruments (14, 18, 24–30) to provide more accurate limb length and recovery of OD. This technique involves careful preoperative planning combined with the intraoperative use of the Horizontal Calibrator plus a double reverse “U” pad. According to a review of the literature (12–14, 16–26, 28–33), no studies on the device have been published. We conducted a prospective controlled study in our hospital to evaluate the efficacy of this technique in reducing LLD and restoring OD after THA.

## Materials and Methods

### Patients

This study was a prospective controlled study. Since there were no blind surgeons, so the use of a single blind. Due to measurement tool design, production, validation, and other reasons, the study was not randomized, yet was grouped according to the time of admission. All patients were informed of the risks and benefits of the trial, gave their consent and signed an informed consent. Inclusion criteria: hip osteoarthritis, development displasia hip, osteonecrosis of the femoral head. Exclusion criteria: proximal femur/acetabular fracture, hemorrhage, malignant tumor, local infection, lower limb bone dysplasia, scoliosis, hip revision, or body intolerance to surgery. This study was approved by the Ethics Committee of Liaoning Provincial People's Hospital and was registered in the Chinese Clinical Trial Registry (ChiCTR2000038040) on 09/09/2020, retrospectively registered. Between 2019 and 2021, we collected 89 THAs, all performed by the same orthopedic surgeon. All acetabular prostheses were Trilogy IT (Zimmer, Warsaw, In, USA), and all femoral prostheses were M\L Taper (Zimmer, Warsaw, In, USA), with a modular head. The choice of femoral prosthesis affects the judgment of limb length and femoral offset during operation. Different types of implants have different penetration depths and femoral offset, so the type and manufacturer of femoral implants need to be controlled so that they are not variable factors.

### Measuring Technique

**Figure 1:**
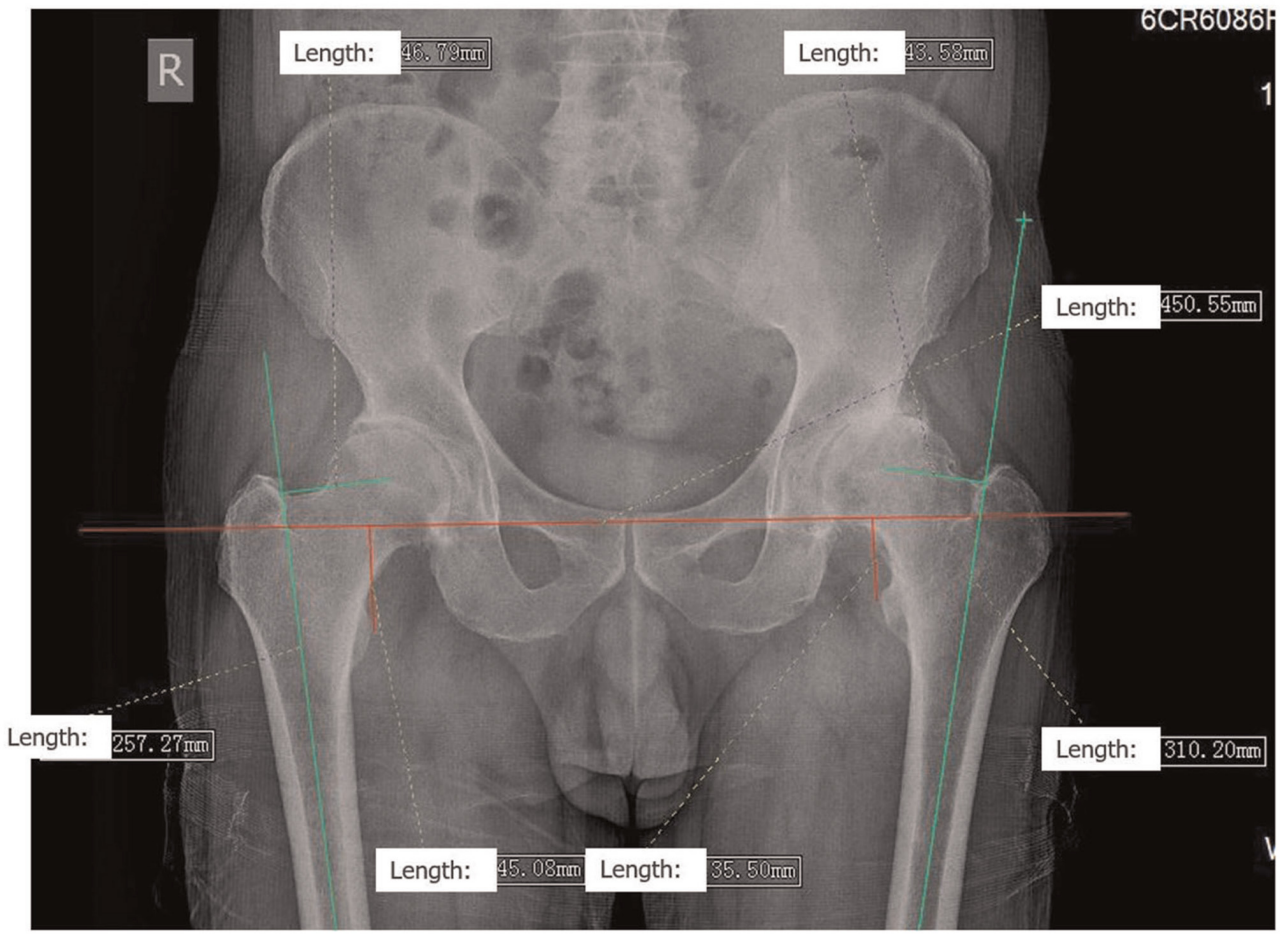
Preoperative X-ray templating All data measurements were made on Neusoft PACS. An anteroposterior (AP) positive view of the pelvis was obtained with both lower extremities internally rotated at 15°. For LLD measurement, a reliable method is to measure the vertical distance from the line connecting the lower edge of the two teardrops to the innermost edge of the small rotor. The difference between the measured values of the two sides is the LLD. OD is measured by the distance between the axis of the femur and the center of the femoral head. In the figure, the LLD was 9.58 mm, the OD on the right was 46.79 mm, and the OD on the left was 43.58 mm.

Radiological examination included an anteroposterior pelvic radiograph with 15° internal rotation of both lower extremities. Depending on the results of the physical examination, a lateral radiograph of the hip and plain radiographs of the spine from other perspectives may be required to detect rigid scoliosis. For LLD measurement, a reliable method is used to measure the vertical distance from the line connecting the lower edge of the two tear drops to the innermost edge of the small rotor. The difference between the measured values of the two sides is the LLD (10). OD is measured by the distance between the axis of femur and the center of the femoral head (Figure 1) (1, 12). To achieve the accuracy of the measurement, all the measurements were carried out on Neusoft PACS (Neusoft Corp., China).

**Figure 2:**
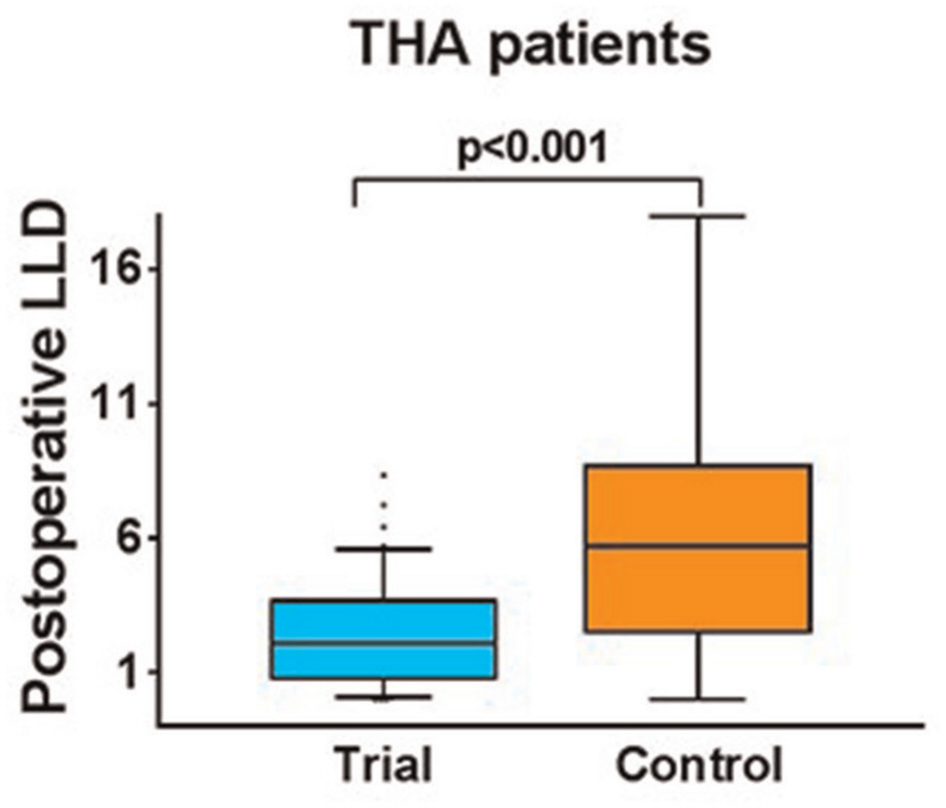
Comparison of postoperative LLD between group I and group II.

### Surgical Technique

**Figure 3:**
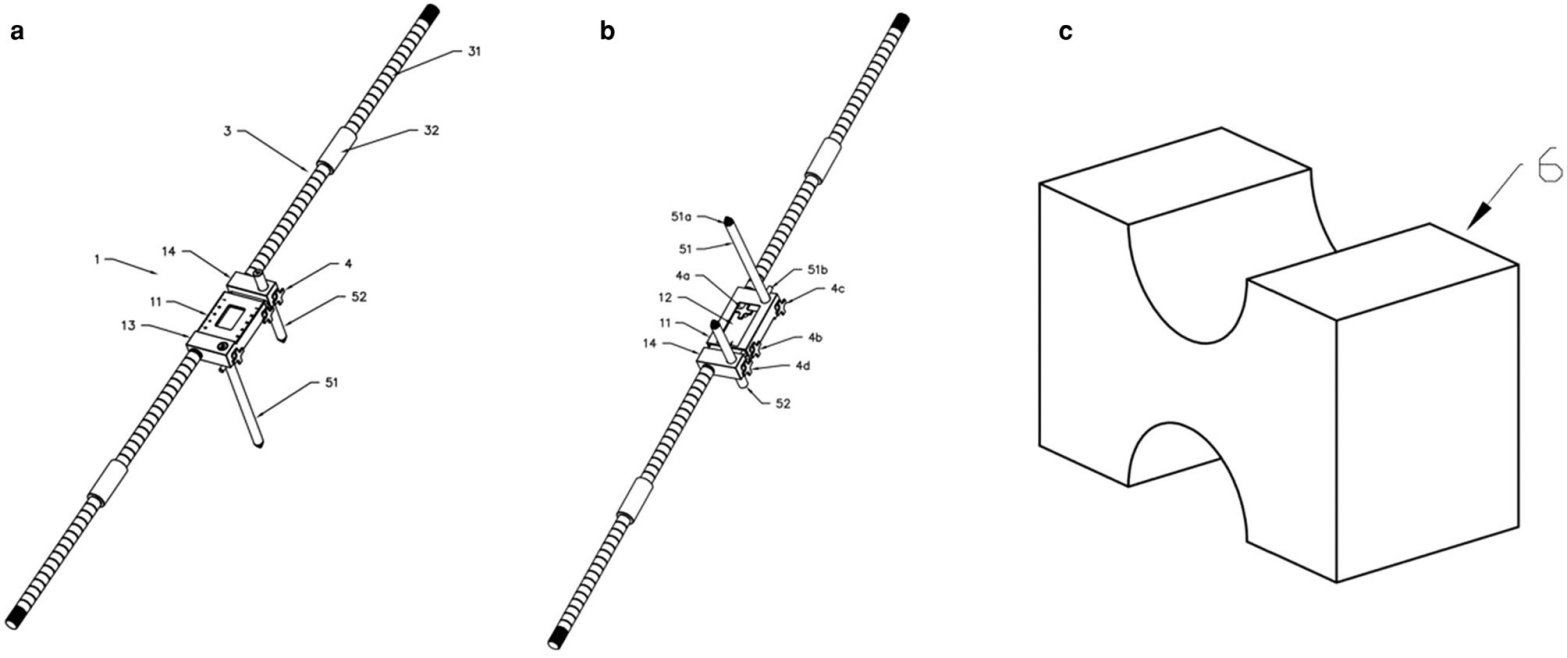
Structure diagram of the horizontal calibrator **(a)** is the front structure diagram of the horizontal calibrator, and **(b)** is the reverse structure diagram. 1—Sliding Scale, 2—Bubble Level, 3—Extension Rod, 4—Lock Screw, 5—Steinman pin, 6—Limb Fixture; 11—first main ruler, 12—second main ruler; 13—First connecting part, 14—Second connecting part;111−0–6 cm ruler scale, 112—rectangular groove, 113—open accommodating groove, 114-lock hole; 121−6–12 cm ruler scale, 122—lock hole groove; 131—first horizontal hole groove, 132—first three-way hole; 141—second horizontal hole groove, 142—second three-way hole; 31—extension rod, 32—connecting cylinder; 41—Threaded joint, 42—Holding part; 51—Lateral pelvic long Steinman pin, 52—Femoral Steinman pin. **(c)** Double reverse “U” pad.

**Figure 4:**
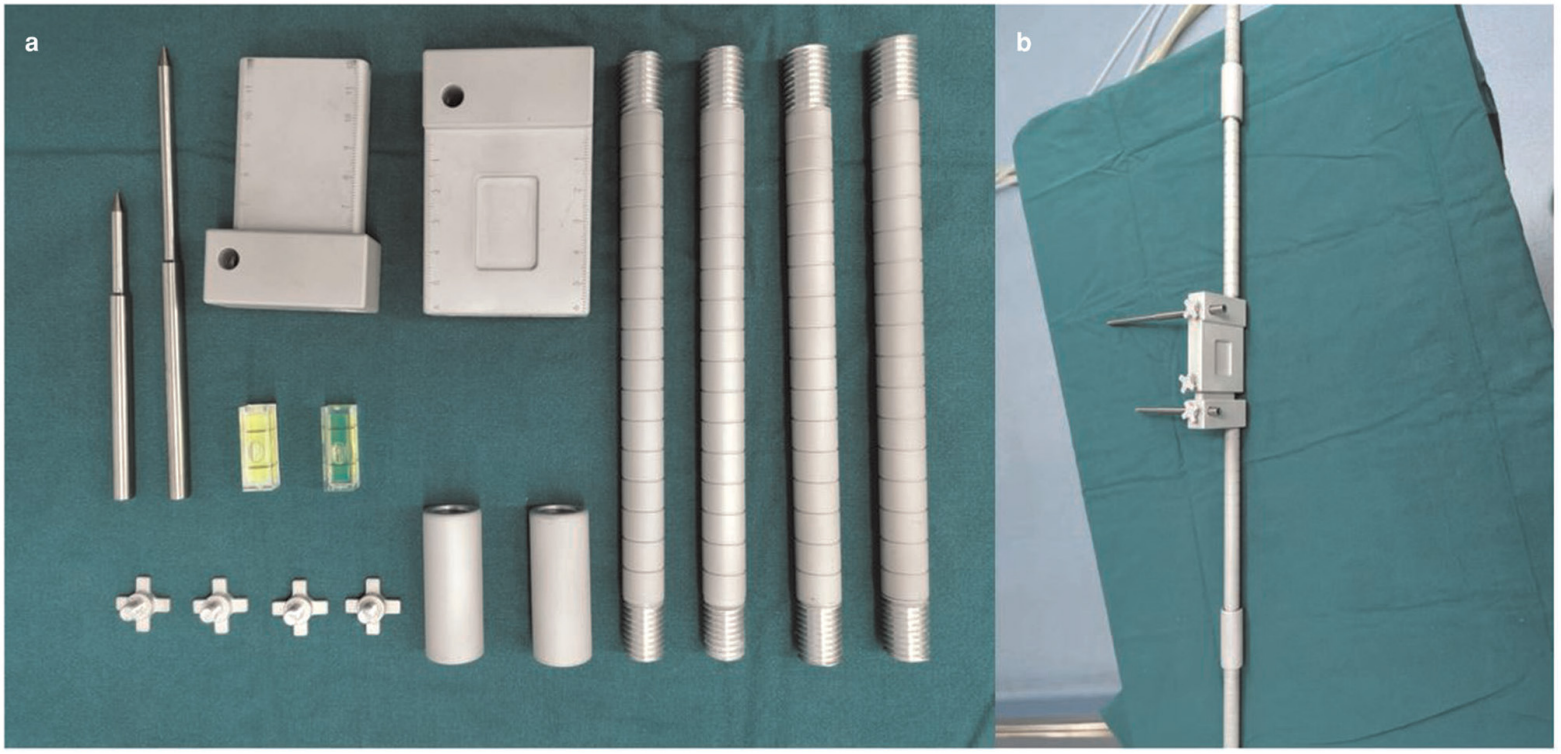
Physical diagram of the horizontal calibrator. **(a)** Shows all the components of the horizontal calibrator. See Figure 3 for details. **(b)** Shows the complete connected physical diagram of the horizontal calibrator, which was reserved preoperatively.

**Figure 5:**
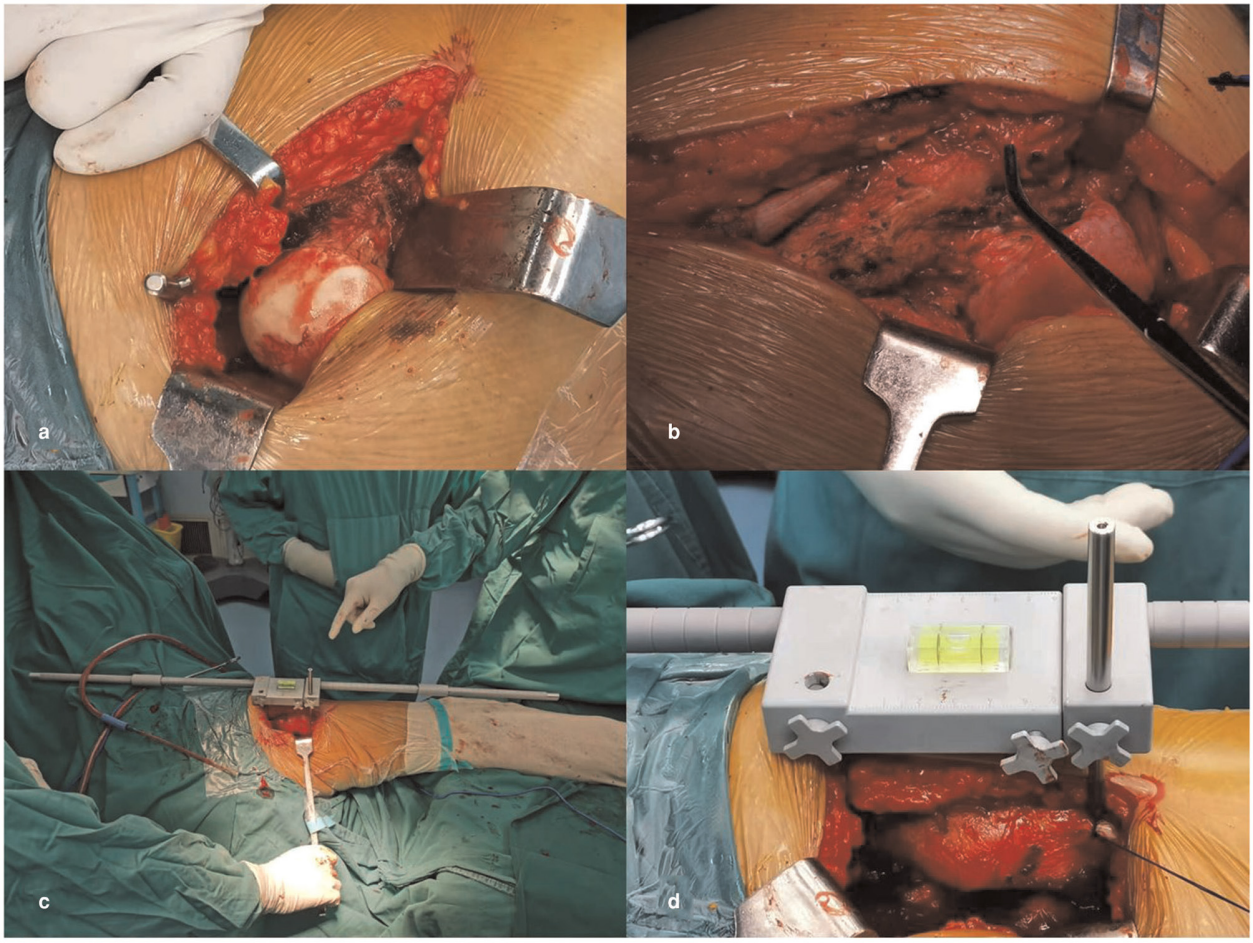
Intraoperative measurement of the horizontal calibrator. A lateral acetabular wire was inserted 3–5 cm above the greater trochanter at 1 o 'clock (right hip) or 11 o 'clock (left hip) as a static reference point in **(a)**. The surgeon looks for a bony projection in the middle of the intertrochanteric spine of the femoral shaft as a reference point in **(b)**. It was made sure that both lower legs are in the grooves of the double reverse “U” pad. The bubble level and extension rod were adjusted so that the bubble of the level is centered and the extension rod is parallel to the affected limb and the longitudinal axis of the body **(c)**. The locking screw was fixed sequentially, the values were read and recorded **(d)**.

To achieve the ideal limb length during the operation, we used the horizontal calibrator plus a double-reverse “U” pad technique to measure the limb length (Figures 3, 4). All operations were performed in the posterior lateral decubitus position. Before the surgery, the double-reverse “U” pad is placed between the legs so that the healthy leg is placed into the lower groove. After sterilizing the towel, the other parts except the acetabular side of the Steinman pin are installed and connected and then set aside (Figure 4b). After exposing and incising the pelvis–trochanter muscles, the limb is placed in an extended position with the affected leg in the upper groove, aligned with the axis of the body and parallel to the ground in order to reproduce this position as much as possible during surgery. At this point, the acetabular side of the Steinman pin was driven into the acetabulum 3–5 cm above the greater trochanter at 1 o'clock (right hip) or 11 o'clock (left hip) as a static reference point (Figure 5a). The surgeon looked for a bony projection in the middle of the intertrochanteric spine of the axis of the femur as a reference point, extending toward the most lateral part of the greater trochanter perpendicular to the axis of the femur for diathermic or suture mark (Figure 5b). The first connection is linked to the upper pelvis side of the Steinman pin, and then another Steinman pin is fixed to the second connection at the femoral side using the marker as a reference. The surgeon reconfirmed that both the legs were in the grooves of the double reverse “U” pad. The bubble level and extension rod were adjusted so that the bubble of the level is centered and the extension rod is parallel to the affected limb and the longitudinal axis of the body (Figure 5c). The locking screw was fixed sequentially, and the values were read and recorded (Figure 5d). The other parts except the lateral acetabular wire were removed and placed aside without disassembling, and the operation was continued. After the installation of the test model, the measurement according to the above steps was repeated. If the limb needs to be lengthened or shortened, it can be adjusted directly according to the preoperative plan. The operative key is to select the correct size of the combination of the femoral component and the modular head so that this distance will be exactly or as close as possible to the differential length measured preoperatively.

### Data Collection and Analysis

The basic information of the patients, the operation time, the anteroposterior radiographs of the pelvis before and 1–6 weeks after the operation were collected, and the femoral deviation and the difference and the LLD were measured and calculated (Figure 1). Pelvic radiographs were reviewed within 6 weeks after surgery because limb length had not been compensated and muscle strength had not been fully restored 1–6 weeks after surgery. Therefore, the data obtained are relatively real, avoiding the influence of other factors. All difference in the measurements were recorded as absolute values; however, compared to the opposite side, the range of values also includes negative values for shortening and positive values for lengthening. Two independent observers recorded all the data before and after the operation, respectively, and the final data was the average of the data recorded by them, which was statistically calculated by the third independent observer. SPSS 23.0(IBM Corp., USA) software was used for independent sample *T*-test for statistical analysis.

## Results

A total of 44 cases of THA were involved in Group I. About 59% of the patients were female, whose age and body mass index were 58.6 ± 9.4 years and 25.3 ± 3.3 kg/m^2^, respectively. A total of 45 THAs were included in Group II, 78% of the patients were female, whose age and body mass index were 61.1 ± 11.5 years and 24 ± 3.4 kg/m^2^, respectively. Age of both (*p* = 0.483; 95%CI = −6.9, 2.0) and body mass index (*p* = 0.979; 95%CI = −0.1, 2.7) were not statistically significant. The operative time of Group I and Group II (*p* = 0.08; 95%CI = −5.7, 12.9) were 83.4 ± 24.3 min and 79.8 ± 19.5 min, respectively, without statistical significance (Table 1). The preoperative radiographic LLD of Group I (mean ± SD = 9.6 ± 7.1 mm, range −51.0 to 19.7 mm) and Group II (mean ± SD = 10.0 ± 8.8 mm, range −21.9 to 32.0 mm) were examined by the independent *T*-test, and the data (*p* = 0.686; 95%CI = −3.8, 3.0) showed no statistically significant difference. There were 14 THAs with LLD <5 mm in Group I preoperatively, accounting for 32%. There were 29 cases with LLD <10 mm, accounting for 66%. In Group II, there were 17 cases with LLD <5 mm before operation, accounting for 38%. There were 26 cases with LLD <10 mm, accounting for 58%. Therefore, there was no significant difference in preoperative radiographic LLD between Group I and Group II. Postoperative LLD of Group I and Group II were 2.5 ± 2.1 mm (range −5.7 to 8.3 mm) and 6.2 ± 4.3 mm (range −18.0 to 15.2 mm), respectively. Independent *t-*test data of the two groups (*p* < 0.001; 95% CI = −5.1, −2.2) showed statistical significance (Figure 2). In Group I, 38 THAs with LLD <5 mm, covering for 86% and LLD <10 mm in 44 cases, covering for 100% were observed. In Group II after surgery, there were 20 THAs in LLD <5 mm, accounting for 44% and there were 36 cases with LLD <10 mm, accounting for 80% (Table 2).

**Table 1:** Baseline table of patients.

**Types**	**Group I** **(*n =* 44)**	**Group II** **(*n =* 45)**	**p**	**95%CI**
Age (years)	58.61± 9.37	61.07± 11.54	0.483	−6.89, 1.98
Female (%)	59%	78%		
Body mass index (Kg/m^2^)	25.27 ± 3.32	24± 3.35	0.979	−0.14, 2.67
Operation time (minutes)	83.39± 24.3	79.8± 19.52	0.08	−5.69, 12.86

**Table 2:** Comparison of radiographic LLD.

**Groups**	**Mean ±SD (mm)**	**Range (mm)**	**<5 mm**	**<10 mm**	***P***	**95%CI**
Preoperative Group I	9.55 ± 7.05	−51.03–19.68	*n =* 14(32%)	*n =* 29(66%)	0.686	−3.76, 2.95
Preoperative Group II	9.95 ± 8.76	−21.94–32	*n =* 17(38%)	*n =* 26(58%)		
Postoperative Group I	2.51 ± 2.09	−5.71–8.34	*n =* 38(86%)	*n =* 44(100%)	<0.001	−5.07, −2.21
Postoperative Group II	6.15 ± 4.29	−17.98–15.16	*n =* 20(44%)	*n =* 36(80%)		

After independent *T*-test of preoperative imaging OD of Group I (mean ± SD: 36.3 ± 6.2 mm, range 23.8–48.5 mm) and Group II (mean± SD: 36.3 ± 9.0 mm, range 17.8–57.3 mm), the data (*p* = 0.052; 95%CI = −3.2, 3.3) was not statistically significant. The imaging OD of Group I and Group II were (mean ± SD: 42.0 ± 6.1 mm, range 31.0–53.1 mm) and (mean ± SD: 43.9 ± 6.8 mm, range 30.4–56.7 mm), respectively. The independent *t-*test data of the two groups (*p* = 0.548; 95%CI = −4.6, 0.8) showed no statistical significance. In addition, we also conducted independent *t-*test analysis of the OD difference before and after surgery. Results of Group I (mean ± SD: 7.6 ± 5.9 mm, range −17.5 to 21.3 mm) and Group II (mean ± SD: 9.4 ± 7.1 mm, range −9.8 to 29.0 mm) showed no statistical significance (*p* = 0.171; 95%CI = −4.6, 1.0) (Table 3).

**Table 3:** Comparison of radiographic OD.

**Groups**	**Mean ±SD (mm)**	**Range (mm)**	***P***	**95%CI**
Preoperative Group I	36.29 ± 6.2	23.8–48.45	0.052	−3.23, 3.29
Preoperative Group II	36.26 ± 8.99	17.8–57.27		
Postoperative Group I	41.99 ± 6.1	30.98–53.1	0.548	−4.62, 0.82
Postoperative Group II	43.89 ± 6.79	30.44–56.73		
Difference Group I	7.61 ± 5.94	−17.47–21.29	0.171	−4.56, 0.97
Difference Group II	9.4 ± 7.12	−9.78–28.99		

## Discussion

In our design, several aspects stand out. First, the design of the double-reverse “U” shape pad is added to solve the problem that the body position is susceptible to change during the measurement process. It refers to the idea of adding a cotton pad between the two legs by Huddleston (24). This design only replaces the bench that is normally placed between the calves and does not increase the time and complexity of the operation. Second, the position of the reference points on the pelvis and the femur is different, and the line between the two reference points is generally not parallel to the extension axis of the limb. Therefore, the limb elongation determined by previous methods does not match the real limb elongation. In our design, extension bars are added on both sides of the calibrator, which can effectively keep the limb in the same horizontal position during the intraoperative measurement. The extension rod has been attached before the skin is cut and does not increase the time spent in the position. Shiramizu et al. (18) also expressed similar views. Third, to avoid changes in the position of the pelvis and the femur side, we chose the two points as fixed points. On the pelvic side, the insertion point of the Steinman pin was selected at 1 o'clock (right hip) or 11 o'clock (left hip) of the acetabulum, and it was 3–5 cm above the greater trochanter, which was close to the center of hip rotation. Many studies have shown that the closer the acetabular measurement point is to the center of rotation, the smaller the measurement error (13, 34). Ranawat et al. (25) also described placing the Steinmann pin vertically in the groove below the ischium of the acetabulum and thus close to the center of rotation. This has been shown to reduce measurement error, even though small changes in limb position, result in a mean postoperative LLD of 2.6 mm. The selected point on the femoral side is a bony process in the middle of the intertrochanteric spine as the reference point and the intersection point extending to the most lateral part of the greater trochanter perpendicular to the axis of the femur. Fourth, in the sliding body part of the calibrator, we have a scale with an accuracy of 1 mm so that the increased or decreased limb length during the operation can be directly viewed, and also the measurement process can be fully quantified. LOOD device described by Barbier et al. (14) and L-shaped calipers described by Shiramizu et al. (18), although they also have scales, have a poor accuracy and can only roughly estimate the measured length. Fifth, a bubble level is placed above the main body of the calibrator so that the calibrator is always parallel to the ground during the measurement process, which adds a second insurance for the measurement. Rice et al. (30) also described that the horizontal bubble meter can make the measurement more accurate. Sixth, to avoid the loosening of the needle, we added a thread at the end. Takigami et al. (19) adopted a triangular-shaped design with double needles and screw ends to avoid intraoperative loosening of the measuring device but at the same time increased surgical trauma.

Preoperative planning and surgical precision are important factors for THA success. How to minimize LLD while maintaining hip stability is a common challenge. Many studies (11, 13, 14, 16–25, 27–37) have been published to describe techniques for LLD management. Freehand techniques are widely used, including intraoperative clinical assessment of soft tissue tension and comparison with contralateral leg position. The literature has identified 933 cases of primary THA in which the freehand technique was used for intraoperative limb balance, with a mean postoperative LLD of 4.42 mm (16). Intraoperative procedures assessing soft tissue tension as an indicator of limb length, such as the Shuck or Dropkick test, may be biased by the patient's position or even the type of anesthesia (38). The dependent position is reproduced when compared to the contralateral leg, and a single palpation marker through asepsis may be inaccurate (39).

The method (12–14, 16–26, 28–33) based on the change of the position of the reference point of the pelvis and femur is an effective way to minimize LLD. A review by Desai et al. (4) concluded that intraoperative calipers combined with preoperative templates is a reliable method to overcome postoperative LLD after THA. The average LLD calculated using intraoperative calipers in literature was 2.89 mm. Shiramizu et al. (18) reported straight calipers and improved L-calipers, and conducted a prospective study of 100 THAs. The results showed that the mean value of L-calipers group was 1.7 ± 1.6 mm, and the mean value of the straight calipers group was 6.2 ± 4.1 mm. Enke et al. (16) made a single incision on the ilium and the most lateral margin of the greater trochanter as a reference point, and conducted a retrospective study of 101 cases of unilateral THA. The results showed that the mean absolute difference (LLD) of the leg length after surgery was 2.51 mm, and the mean deviation difference was 2.39 mm. Nevertheless, this method is far from the center of acetabular rotation, which increases measurement error and additional trauma. Gupta and Papadopoulos et al. (12, 17) described a double-stitch technology, that is, tie a knot with silk thread on the skin about 10 cm from the proximal end of the great trochanter, and then draw the other end with vascular forceps to make a diathermic mark on the most lateral edge of the great trochanter. They performed 60 THAs using this technique and showed a mean postoperative LLD of 1.58 mm (range −8 mm to 7 mm). The technique is simple but susceptible to soft tissue and 3D space.

Inadequate OD reduces soft tissue tension and increases the risk of dislocation. Restoring this soft tissue tension by increasing the length of the femoral neck may increase the length of the leg. A large offset increases the risk of rotor bursitis and adduction tendinitis. The average cervical stem angle of adult males was 129.6°(range 113.2°-148.2°) and that of adult females was 131.9°(range 107.1°-151.9°). Patients with cervical stem value significantly lower than this value could have hip varus, and vice versa (3). Woolson et al. (40) argued that excessive leg lengthening to increase stability was unacceptable. Contrary to the rationalization of the theory that inadequate soft tissue tone leads to increased postoperative stability, their study found that patients with short legs did not have an increased incidence of postoperative dislocation compared with patients with long or similar legs. Studies have confirmed that abnormal lever arms increase the wear of polyethylene in the prosthesis, which may lead to aseptic loosening (12). Kurtz (13) described the *in situ* femur measurement technique, which refers to the implantation of the femoral prosthesis before the femoral neck osteotomy without dislocation of the hip joint so that the implanted femoral prosthesis can be used to measure the LLD and OD. Ninety-three patients (100 hips) were treated with this technique and the difference between the *in situ* measurements and the preoperative and postoperative radiographic measurements was a mean leg length of 0.1 mm and a OD of 0.4 mm. This method is as close as possible to the rotation center of the hip joint, and the fixation pins on the acetabulum are not easy to come off. But the technology's complexity has limited its widespread use. Although our technique did not show statistically significant differences in the recovery of OD, our Group I patients performed better than Group II after surgery. Next, we will continue to improve the device by installing a vortex-like structure on the measuring body to accurately measure the femoral offset.

There are some flaws in our study. First of all, the outcome indicators in our study only collected imaging and basic patient information, without measuring structural limb length and functional scoring. We simply pursued the absolute equality of the limbs in imaging, ignoring the patient's feelings. The study of Fujita et al. (5) showed that patients with little or no LLD after THA still felt uncomfortable with their leg length due to residual pelvic inclination. Secondly, although we added extension rods and double-reverse “U” shaped pads to ensure that the patient's position did not change, for some obese patients, it was still not possible to ensure that their position did not change during the operation because the side stopper could not be fixed. Finally, our technique still requires a learning curve, adding 3–5 min to the traditional method.

## Conclusion

The horizontal calibrator can provide more accurate limb length and femoral offset recovery in THA. It is a simple surgical technique that does not add additional surgical costs and does not significantly increase operative time, providing a new solution for surgeons to resolve postoperative LLD and restore femoral offset.

## Data Availability Statement

The original contributions presented in the study are included in the article/supplementary material, further inquiries can be directed to the corresponding author/s.

## Ethics Statement

The studies involving human participants were reviewed and approved by the Ethics Committee of Liaoning Provincial People's Hospital. The patients/participants provided their written informed consent to participate in this study.

## Author Contributions

All authors listed have made a substantial, direct, and intellectual contribution to the work and approved it for publication.

## Funding

This work was supported by the National Natural Science Foundation of China (Grant No. 81671811) and Shenyang Science and Technology Innovation Platform Construction Plan (Grant No. 1800975).

## Conflict of Interest

The authors declare that the research was conducted in the absence of any commercial or financial relationships that could be construed as a potential conflict of interest.

## Publisher's Note

All claims expressed in this article are solely those of the authors and do not necessarily represent those of their affiliated organizations, or those of the publisher, the editors and the reviewers. Any product that may be evaluated in this article, or claim that may be made by its manufacturer, is not guaranteed or endorsed by the publisher.

## References

[1] Sculco PK Austin MS Lavernia CJ Rosenberg AG Sierra RJ. Preventing leg length discrepancy and instability after total hip arthroplasty. Instr Course Lect. 65:225–41 (2016). 27049193

[2] Bokshan SL Ruttiman RJ DePasse JM Eltorai AEM Rubin LE Palumbo MA Daniels AH. Reported litigation associated with primary hip and knee arthroplasty. J Arthroplasty. (2017) 32:3573–7 e3571. 10.1016/j.arth.2017.07.001 28781019

[3] Kayani B Pietrzak J Hossain FS Konan S Haddad FS. Prevention of limb length discrepancy in total hip arthroplasty. Br J Hosp Med. (2017) 78:385–90. 10.12968/hmed.2017.78.7.385 28692359

[4] Desai AS Dramis A Board TN. Leg length discrepancy after total hip arthroplasty: a review of literature. Curr Rev Musculoskelet Med. (2013) 6:336–41. 10.1007/s12178-013-9180-0 23900834 PMC4094096

[5] Fujita K Kabata T Kajino Y Tsuchiya H. Optimizing leg length correction in total hip arthroplasty. Int Orthop. (2020) 44:437–43. 10.1007/s00264-019-04411-0 31595310

[6] Nossa JM Munoz JM Riveros EA Rueda G Marquez D Perez J. Leg length discrepancy after total hip arthroplasty: comparison of 3 intraoperative measurement methods. Hip Int. (2018) 28:254–8. 10.5301/hipint.5000577 29192732

[7] Grobler G Nortje M Dower B Chivers D. A Vertical measurement system to predict the change in leg length in total hip arthroplasty. Arthroplasty today. (2020) 6:330–7. 10.1016/j.artd.2020.04.008 32514423 PMC7267683

[8] Jassim SS Ingham C Keeling M Wimhurst JA. Digital templating facilitates accurate leg length correction in total hip arthroplasty. Acta Orthopaedica Belgica. (2012) 78:344–9 22822575

[9] Knight JL Atwater RD. Preoperative planning for total hip arthroplasty. Quantitating its utility and precision. J Arthroplasty. (1992) 7:403–9. 10.1016/S0883-5403(07)80031-3 1431923

[10] Debbi EM Rajaee SS Mayeda BF Penenberg BL. Determining and achieving target limb length and offset in total hip arthroplasty using intraoperative digital radiography. J Arthroplasty. (2020) 35:779–85. 10.1016/j.arth.2019.10.003 31699530

[11] Ezzet KA McCauley JC. Use of intraoperative X-rays to optimize component position and leg length during total hip arthroplasty. J Arthroplasty. (2014) 29:580–5. 10.1016/j.arth.2013.08.003 24074889

[12] Papadopoulos DV Koulouvaris P Aggelidakis GC Tsantes AG Lykissas MG Mavrodontidis A. Intraoperative measurement of limb lengthening during total hip arthroplasty. Indian J Orthop. (2017) 51:162–7. 10.4103/0019-5413.201716 28400661 PMC5361466

[13] Kurtz WB. In situ leg length measurement technique in hip arthroplasty. J Arthroplasty. (2012) 27:66–73. 10.1016/j.arth.2011.02.003 21435822

[14] Barbier O Ollat D Versier G. Interest of an intraoperative limb-length and offset measurement device in total hip arthroplasty. Orthop Traumatol Surg Res. (2012) 98:398–404. 10.1016/j.otsr.2012.02.004 22560790

[15] Fackler CD Poss R. Dislocation in total hip arthroplasties. Clin Orthop Relat Res. (1980) (151):169–78. 10.1097/00003086-198009000-00023 7418301

[16] Enke O Levy YD Bruce WJ. Accuracy of leg length and femoral offset restoration after total hip arthroplasty with the utilisation of an intraoperative calibration gauge. Hip Int. (2020) 30:296–302. 10.1177/1120700019836383 30924374

[17] Gupta R Pathak P Singh R Majumdar KP. Double-stitch technique: a simple and effective method to minimize limb length discrepancy after total hip arthroplasty. Indian J Orthop. (2019) 53:169–73. 10.4103/ortho.IJOrtho_188_18 30905998 PMC6394180

[18] Shiramizu K Naito M Shitama T Nakamura Y Shitama H. L-shaped caliper for limb length measurement during total hip arthroplasty. J Bone Joint Surg Br Vol. (2004) 86:966–9. 10.1302/0301-620X.86B7.14587 15446519

[19] Takigami I Itokazu M Itoh Y Matsumoto K Yamamoto T Shimizu K. Limb-length measurement in total hip arthroplasty using a calipers dual pin retractor. Bull NYU Hosp Joint Dis. 66:107–10 (2008) 18537779

[20] Knight WE. Accurate determination of leg lengths during total hip replacement. Clin Orthop Relat Res. (1977) 123:27–8. 10.1097/00003086-197703000-00010 852182

[21] Bose WJ. Accurate limb-length equalization during total hip arthroplasty. Orthopedics. (2000) 23:433–6 10825109

[22] Desai AS Connors L Board TN. Functional and radiological evaluation of a simple intra operative technique to avoid limb length discrepancy in total hip arthroplasty. Hip Int. (2011) 21:192–8. 10.5301/hip.2011.6514 21484733

[23] Woolson ST Harris WH. A method of intraoperative limb length measurement in total hip arthroplasty. Clin Orthop Relat Res. (1985) 194:207–10. 10.1097/00003086-198504000-00032 3978918

[24] Huddleston HD. An accurate method for measuring leg length and hip offset in hip arthroplasty. Orthopedics. (1997) 20:331–2. 10.3928/0147-7447-19970401-10 9127867

[25] Ranawat CS Rao RR Rodriguez JA Bhende HS. Correction of limb-length inequality during total hip arthroplasty. J Arthroplasty. (2001) 16:715–20. 10.1054/arth.2001.24442 11547369

[26] Naito M Ogata K Asayama I. Intraoperative limb length measurement in total hip arthroplasty. Int Orthop. (1999) 23:31–3. 10.1007/s002640050298 10192014 PMC3619784

[27] Ng VY Kean JR Glassman AH. Limb-length discrepancy after hip arthroplasty. J Bone Joint Surg Am Vol. (2013) 95:1426–36. 10.2106/JBJS.L.00433 23925749

[28] Desai A Barkatali B Dramis A Board TN. A simple intraoperative technique to avoid limb length discrepancy in total hip arthroplasty. Surgeon. (2010) 8:119–21. 10.1016/j.surge.2009.10.023 20303896

[29] McGee HM Scott JH. A simple method of obtaining equal leg length in total hip arthroplasty. Clin Orthop Relat Res. (1985) (194):269–70. 10.1097/00003086-198504000-00042 3978924

[30] Rice IS Stowell RL Viswanath PC Cortina GJ. Three intraoperative methods to determine limb-length discrepancy in THA. Orthopedics. (2014) 37:e488–495. 10.3928/01477447-20140430-61 24810827

[31] Ranawat CS Rodriguez JA. Functional leg-length inequality following total hip arthroplasty. J Arthroplasty. (1997) 12:359–64. 10.1016/S0883-5403(97)90190-X 9195310

[32] Itokazu M Masuda K Ohno T Itoh Y Takatsu T Wenyi Y. A simple method of intraoperative limb length measurement in total hip arthroplasty. Bulletin 56:204–5 (1997). 9438079

[33] Matsuda K Nakamura S Matsushita T. A simple method to minimize limb-length discrepancy after hip arthroplasty. Acta Orthop. (2006) 77:375–9. 10.1080/17453670610046280 16819674

[34] Tagomori H Kaku N Tabata T Tsumura H. A new and simple intraoperative method for correction of leg-length discrepancy in total hip arthroplasty. J Orthopaedics. (2019) 16:405–8. 10.1016/j.jor.2019.04.007 31193035 PMC6514264

[35] Sarin VK Pratt WR Bradley GW. Accurate femur repositioning is critical during intraoperative total hip arthroplasty length and offset assessment. J Arthroplasty. (2005) 20:887–91. 10.1016/j.arth.2004.07.001 16230240

[36] Halai M Gupta S Gilmour A Bharadwaj R Khan A Holt G. The Exeter technique can lead to a lower incidence of leg-length discrepancy after total hip arthroplasty. Bone Joint J. (2015) 97-B:154–9. 10.1302/0301-620X.97B2.34530 25628275

[37] Alazzawi S Douglas SL Haddad FS. A novel intra-operative technique to achieve accurate leg length and femoral offset during total hip replacement. Ann R Coll Surg Engl. (2012) 94:281–2. 10.1308/rcsann.2012.94.4.281a 22613318 PMC3957519

[38] Sathappan SS Ginat D Patel V Walsh M Jaffe WL Di Cesare PE. Effect of anesthesia type on limb length discrepancy after total hip arthroplasty. J Arthroplasty. (2008) 23:203–9. 10.1016/j.arth.2007.01.022 18280413

[39] Xue E Su Z Chen C Wong PK Wen H Zhang Y. An intraoperative device to restore femoral offset in total hip arthroplasty. J Orthop Surg Res. (2014) 9:58. 10.1186/s13018-014-0058-7 25037492 PMC4223551

[40] Della Valle CJ Di Cesare PE. Complications of total hip arthroplasty: neurovascular injury, leg-length discrepancy, and instability. Bulletin. (2001) 60:134–42. 12102400

