# Corrigendum: Accuracy of the Horizontal Calibrator in Correcting Leg Length and Restoring Femoral Offset in Total Hip Arthroplasty

**DOI:** 10.3389/fsurg.2022.890691

**Published:** 2022-08-04

**Authors:** Xing Chen, Shuxing Xing, Zhiyong Zhu, Huisheng Wang, Zhongshen Yu, Xizhuang Bai, Xi Li

**Affiliations:** ^1^ Department of Orthopedic Surgery, Chengdu Fifth People’s Hospital, The Fifth People’s Hospital of Chengdu University of TCM, Chengdu, China; ^2^ Department of Orthopedics and Sports Medicine and Joint Surgery, Liaoning Provincial People’s Hospital, People92s Hospital of China Medical University, Shenyang, China

**Keywords:** hip arthroplasty, leg length discrepancy, offset, intraoperative, calibrator

A Corrigendum on Accuracy of the Horizontal Calibrator in Correcting Leg Length and Restoring Femoral Offset in Total Hip Arthroplasty by Chen X., Xing S., Zhu Z., Wang H., Yu Z., Bai X., et al. (2022). Front. Surg. 9:845364. doi: 10.3389/fsurg.2022.845364

1. In the original article, there was an error in “MATERIALS AND METHODS “ section, “Measuring Technique” sub-section, Paragraph one, Line 10–12 : “OD is measured by the distance between the axis of femur and the center of the femoral head (Figures 1, 2) (1, 12).”

**Figure 1:**
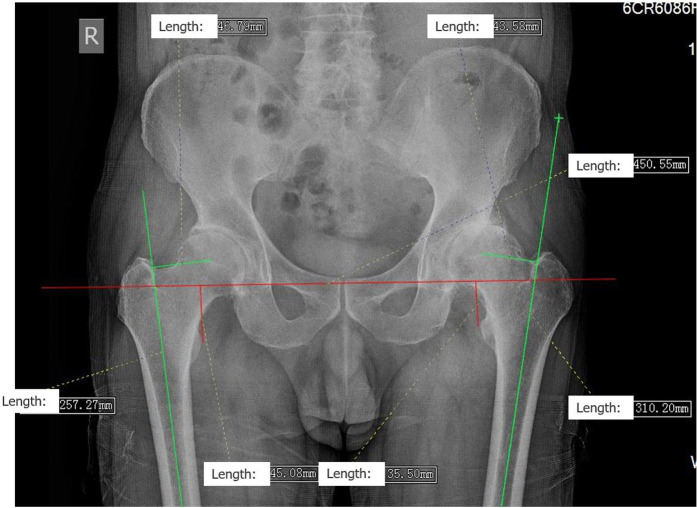


**Figure 2:**
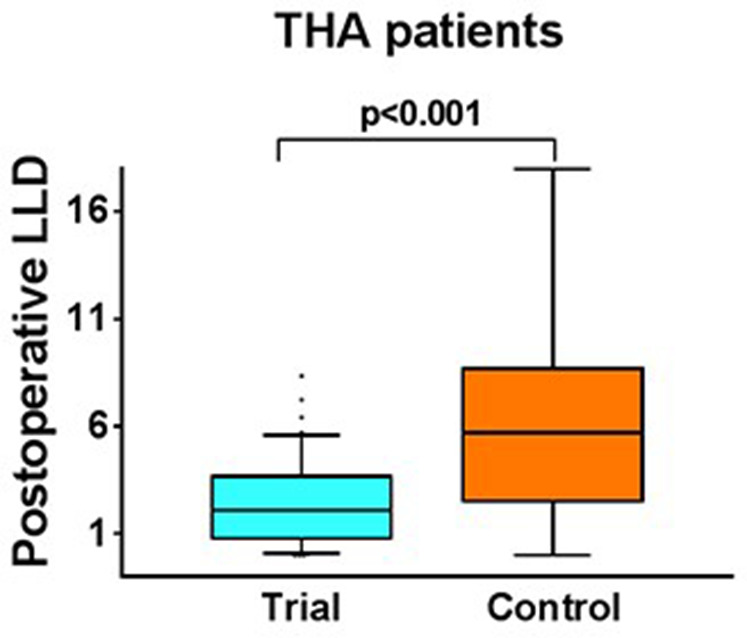


A correction has been made to “MATERIALS AND METHODS “ section, “Measuring Technique” sub-section, Paragraph one, Line 10–12 : “OD is measured by the distance between the axis of femur and the center of the femoral head (Figure 1) (1, 12).”

2. In the original article, there was an error in “RESULTS “ section, Paragraph one, Line 24–26 : “Independent *t*-test data of the two groups (*p* < 0.001; 95% CI = −5.1, −2.2) showed statistical significance (Figure 5).”

**Figure 5:**
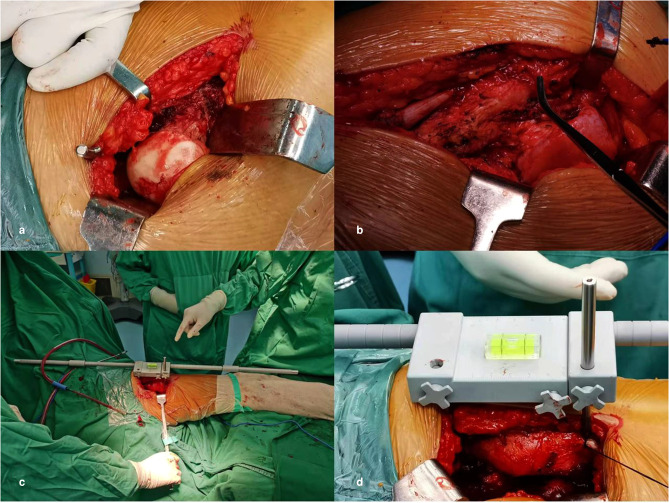


A correction has been made to “RESULTS “section, Paragraph one, Line 24–26 : “Independent *t-*test data of the two groups (*p *< 0.001; 95% CI = −5.1, −2.2) showed statistical significance (Figure 2).”

3. In the original article, there was a mistake in the legend for “FIGURE 4” as published : “Physical diagram of the horizontal calibrator. (**a**) Shows all the components of the horizontal calibrator. See Figure 2 for details. (**b**) Shows the complete connected physical diagram of the horizontal calibrator, which was reserved preoperatively.”

**Figure 4:**
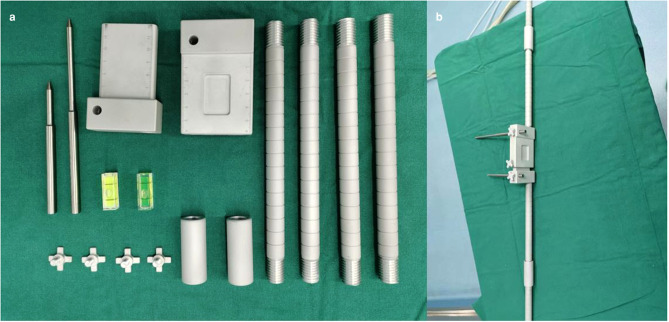


The correct legend appears below:

“Physical diagram of the horizontal calibrator. (**a**) Shows all the components of the horizontal calibrator. See Figure 3 for details. (**b**) Shows the complete connected physical diagram of the horizontal calibrator, which was reserved preoperatively.”

**Figure 3:**
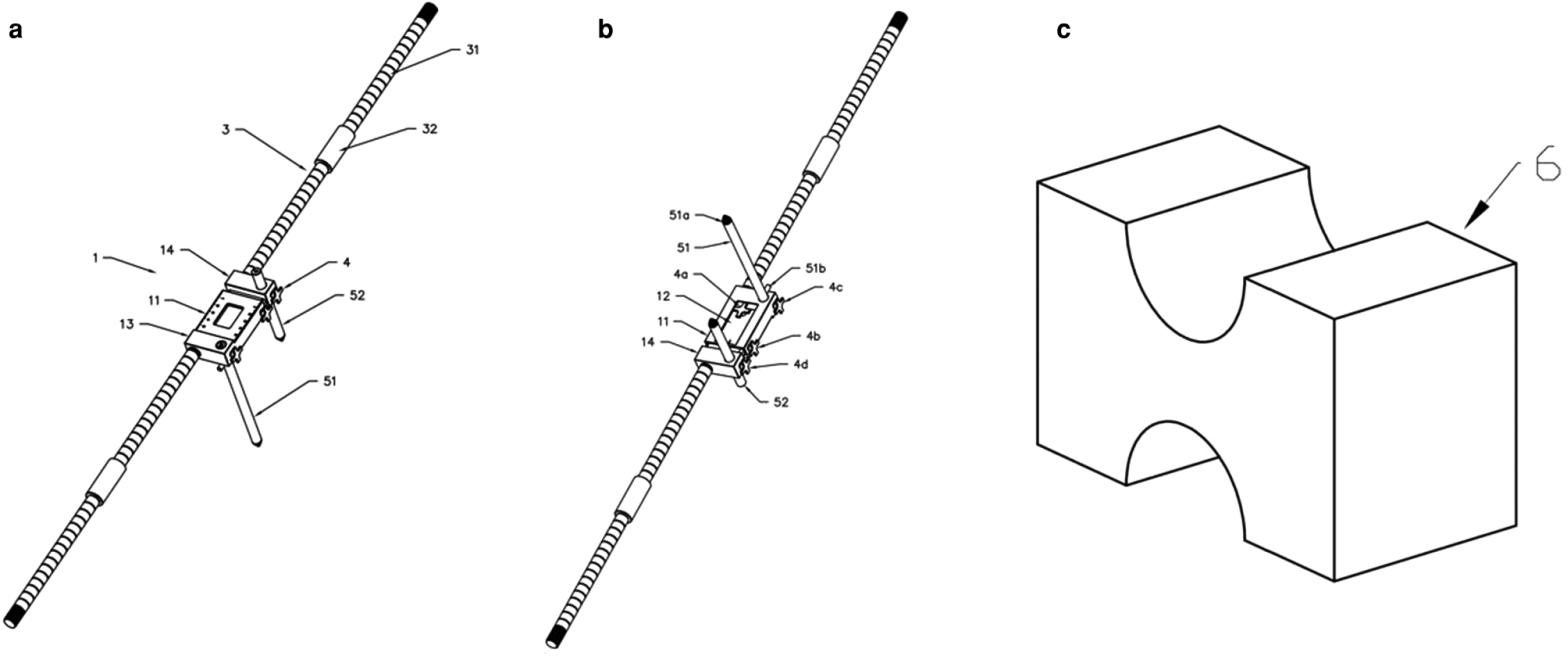


The authors apologize for these errors and state that these do not change the scientific conclusions of the article in any way. The original article has been updated.

